# The Neural Correlates of Chronic Symptoms of Vertigo Proneness in Humans

**DOI:** 10.1371/journal.pone.0152309

**Published:** 2016-04-18

**Authors:** Ola Alsalman, Jan Ost, Robby Vanspauwen, Catherine Blaivie, Dirk De Ridder, Sven Vanneste

**Affiliations:** 1 Lab for Clinical & Integrative Neuroscience, School of Behavioral and Brain Sciences, The University of Texas at Dallas, Dallas, United States of America; 2 BRAI^2^N, Sint Augustinus Hospital Antwerp, Antwerp, Belgium; 3 ENT Department, Sint Augustinus Hospital Antwerp, European Institute for ORL-HNS, Antwerp, Belgium; 4 Department of Surgical Sciences, Section of Neurosurgery, Dunedin School of Medicine, University of Otago, Dunedin, New Zealand; UMR8194, FRANCE

## Abstract

Vestibular signals are of significant importance for variable functions including gaze stabilization, spatial perception, navigation, cognition, and bodily self-consciousness. The vestibular network governs functions that might be impaired in patients affected with vestibular dysfunction. It is currently unclear how different brain regions/networks process vestibular information and integrate the information into a unified spatial percept related to somatosensory awareness and whether people with recurrent balance complaints have a neural signature as a trait affecting their development of chronic symptoms of vertigo. Pivotal evidence points to a vestibular-related brain network in humans that is widely distributed in nature. By using resting state source localized electroencephalography in non-vertiginous state, electrophysiological changes in activity and functional connectivity of 23 patients with balance complaints where chronic symptoms of vertigo and dizziness are among the most common reported complaints are analyzed and compared to healthy subjects. The analyses showed increased alpha2 activity within the posterior cingulate cortex and the precuneues/cuneus and reduced beta3 and gamma activity within the pregenual and subgenual anterior cingulate cortex for the subjects with balance complaints. These electrophysiological variations were correlated with reported chronic symptoms of vertigo intensity. A region of interest analysis found reduced functional connectivity for gamma activity within the vestibular cortex, precuneus, frontal eye field, intra-parietal sulcus, orbitofrontal cortex, and the dorsal anterior cingulate cortex. In addition, there was a positive correlation between chronic symptoms of vertigo intensity and increased alpha-gamma nesting in the left frontal eye field. When compared to healthy subjects, there is evidence of electrophysiological changes in the brain of patients with balance complaints even outside chronic symptoms of vertigo episodes. This suggests that these patients have a neural signature or trait that makes them prone to developing chronic balance problems.

## Introduction

The risk of developing symptoms, such as chronic symptoms of vertigo and dizziness, are increased among the aging population and are often associated with other neurological deficits and chronic medical problems. Symptoms of vertigo are not a disease entity but rather a symptom of various disorders with different causes and pathophysiological mechanisms [1–5]. The current prevalence of vertigo and dizziness is approximately 7.4% in the general population ages 18 to 97 years [6–8], and vestibular disorders are the cause of approximately 50% of cases of chronic balance complaints in the elderly [9, 10]. In addition, it was estimated that 88% of patients complain of recurrent balance problems, resulting in increased frequency of occupational sick leave or recurrent medical consultations [3, 5]. Although vertigo and dizziness are common complaints of those seeking medical attention—particularly the elderly [11, 12]—there continues to be a deficit of knowledge on the subject.

The question arises whether patients with recurrent balance complain with chronic vestibular symptoms have a trait, which makes them prone to developing these symptoms, and if so, whether there exists a neural signature of this proneness in their resting state brain activity and connectivity in between their symptoms.

Vestibular processing occurs in different brain regions, implying a multimodal impairment of sensory integration that involves multiple regions [13]. All natural vestibular stimuli are multimodal, and multiple sensory inputs converge at all levels of the central vestibular system [14]. The vestibular percept of body position and motion is always relative to the subject’s surrounding (i.e. exocentric), whereas the visual and auditory percepts are always relative to the subject within that space (i.e. egocentric) [14]. Furthermore, the vestibular cortical areas are represented in both hemispheres, with an ipsilateral predominance for processing ipsilateral input and clear lateralization: in right handed people the right vestibular cortex is dominant, and the left vestibular cortex is in left handed people [14, 15].

A core vestibular network has been described based on an ALE-meta-analysis, which involves the posterior insula, retroinsular cortex, and parietal operculum. In other words, the superior temporal and inferior parietal cortex are where vestibular afferents converge [16]. The posterior insula and temporo-parietal cortex core area receive input from the thalamus and vestibular stimuli often co-activating the frontal operculum, anterior insula [15, 17–19], the intraparietal sulcus, frontal eye fields, hippocampus and parahippocampal area, anterior cingulate gyrus, and precuneus [16, 20, 21]. These reports demonstrate the level of uncertainty with regard to the exact anatomical identification of a vestibular network, which is likely widely distributed in nature. In addition, in healthy subjects functional connections exist between the abovementioned vestibular areas. For example, research exists supporting a joint vestibular network between the opercullum, temporo-parietal regions, premotor cortex, and the anterior cingulate cortex, according to a concept from animal literature termed the inner vestibular circle [22]. Moreover, combined structural and functional connectivity mapping using diffusion tensor imaging and functional connectivity magnetic resonance in healthy subjects describes a link between the vestibular nuclei and parieto-insular-vestibular cortex [23].

At present, it is neither clear how different regions in the brain process vestibular information, nor how they integrate this information into a global unified vestibular percept. Most of our current understanding of the human vestibular network has been derived from human lesion studies [24, 25] as well as studies in healthy controls [22, 26, 27] including PET studies comparing healthy controls to a patient population [28], complemented by tracer and electrophysiological studies in animals [29–32]. Therefore, the aim of this paper is to verify whether there exists a neural signature of proneness to chronic vestibular symptoms characterized by a pattern of vestibular activation and connectivity in the resting brain (i.e. in-between vestibular spells).

## Materials and Methods

### Participants

Twenty-three patients with chronic balance complaints (*M* = 52.17 years; *Sd* = 12.28; 9 males and 14 females) were included in this analysis. Healthy subjects were excluded if they had a history of neurological illness, psychiatric illness, or the presence of other physical disabilities. All patients with chronic symptoms of vertigo were examined by an otolaryngologist in the ENT Department of the Sint Augustinus Hospital in Antwerp, Belgium. For the healthy controls, a hearing assessment was not performed. Detailed descriptive information and profiles of patients with balance complaints can be found in Tables 1 & 2.

**Table 1:** Specific characteristics of patients’ balance complaints and it accompanying symptoms.

Characteristic	# of subjects	Vestibular symptoms	Non-vestibular symptoms
**Duration**		Diminished concentration	Phonophobia
Minutes	(n = 1)	Aural fullness	Anorexia
Hours	(n = 1)	Brain thrombosis	Neck problems
Days	(n = 3)	Amblyopia	Sleeping disorders
Continuous	(n = 8)	Pressure in head	Hypothyroid
Not known	(n = 9)	Photophobia	Low blood pressure
**Frequency**		Visual vertigo	Hyperventilation
Daily	(n = 13)	Hearing loss	Sleeping feet
Weekly	(n = 1)	Blurry vision	
Not known	(n = 9)	Autophonia	
**Type**		Diplopia	
Vertigo	(n = 23)	Dizziness	
Gait disorder	(n = 11)	Somnolence	
Nausea	(n = 11)	Motion sickness	
		Oscillopsia	
		Facial nerve complaints	
		Headache	
		Photophobia	
		Cervicalgia	
		Briachialgia	
		Space discomfort	
		Tinnitus	
		Migraine	

**Table 2:** Profile of patients with chronic vestibular symptoms. The cervical-vestibular-evoked myogenic potential (cVEMP), the vestibular electronystagmography (vENG -caloric irrigation), the hospital anxiety and depression scale (HADS): (0–7) mild anxiety/depression, (8–10) possible anxiety/depression, (11–21) probable anxiety/depression., the dizziness and handicap inventory (DHI): (0–29) mild, (30–59) moderate, (60–100) severe., and the hyperventilation (Nijmegen) scores: from score 18 hyperventilation is possible, from score 23 hyperventilation for 80% sure.

	cVEMP	vENG -caloric irrigation	HADS-A	HADS-D	DHI	Hyperventilation(Nijmegen)
1	Bilateral absent response	unknown	7	4	72	34
2	Symmetrical	Symmetrical	11	7	48	14
3	Unknown	Symmetrical	6	8	50	12
4	Areflexive on right side	Canal hypofunction on left side (39%)	4	7	50	15
5	Areflexive on left side	Canal hypofunction on left side (61%)	6	3	38	22
6	Symmetrical	Symmetrical	13	15	66	Unknown
7	Unknown	Symmetrical	15	9	68	Unknown
8	Symmetrical	Canal hypofunction on left side (62%)	9	3	28	12
9	areflexive on left side	Symmetrical	10	12	56	29
10	Bilateral absent response	Symmetrical	4	1	16	Unknown
11	Unknown	Unknown	11	5	38	Unknown
12	Otolith hypofunction on left side	Symmetrical	12	8	42	24
13	Areflexive on left side	Unknown	6	3	64	Unknown
14	Areflexive on left side	Canal hypofunction on left side (66%)	0	7	34	6
15	Unknown	Canal hypofunction on left side (88%)	10	4	48	21
16	Symmetrical	Symmetrical	3	1	48	7
17	Symmetrical	Canal hypofunction on left side (22%)	2	5	40	7
18	Symmetrical	Canal hypofunction on left side (23%)	7	12	64	18
19	Symmetrical	Symmetrical	7	5	68	33
20	symmetrical	Symmetrical	10	8	54	33
21	Otolith hypofunction on right side	Symmetrical	6	9	46	11
22	Unknown	Canal hypofunction on left side (100%)	3	2	46	7
23	Otolith hypofunction on left side	Canal hypofunction on left side (46%)	11	10	46	22

### Healthy control group

Group age and gender matched EEG data of a healthy control group (*N* = 23; *M* = 51. 47 years; *Sd* = 11.58; 10 males, and 13 females) was collected. None of the healthy subjects reported any history of chronic symptoms of vertigo. Exclusion criteria included known psychiatric or neurological illness, psychiatric history, drug/alcohol abuse, history of head injury (with loss of consciousness), seizures, headache, or physical disability. This study was approved by the local ethical committee (Sint Augustinus Hospital Antwerp) and was in accordance with the declaration of Helsinki. Collection of the data was under approval of IRB GZA OGA85 all patients gave written informed consent.

### Questionnaires and vestibular testing

Visual analogue scale (VAS). The VAS for intensity and discomfort was assessed at baseline. Using the VAS, the patient marks on a continuum line (10cm) the point that they feel represents the perception of their current state [33, 34]. The VAS asked patients to specify the amount of intensity and discomfort of their vestibular symptoms in a continuum scale from (0 = not intense) to (10 = as intense as imaginable), and (0 = no discomfort) to (10 = as much discomfort as imaginable).

Dizziness handicap inventory (DHI). The purpose of the DHI is to quantify the degree of handicap that patients experience due to their balance complaints. The DHI quantifies for the emotional, physical, or functional handicap s that patients experience [35]. Scores ranges from 0–100 with scores categorized as follow: 0–29 (= mild handicap), 30–59 (= moderate handicap), and 60–100 (= severe handicap).

Hospital anxiety and depression scale (HADS). The HADS is a self-assessment scale, developed to detect states of depression, anxiety and emotional distress amongst patients who were being treated for a variety of clinical problems [36]. Scores ranges from 0–21 with scores categorized as follow: 0–7 (= no anxiety/depression), 8–10 (= possible anxiety/depression), and 11–21 (= probable anxiety/depression).

Cervical-vestibular-evoked myogenic potential (cVEMP). Is recorded from the surface of sternocelidomastiod muscle (SCM) and generated by the activation of saccular afferents, reflects the function of the saccule and inferior vestibular nerve [37].

Caloric irrigation test (vENG). Is part of the electronystagmography test that aims to identify the degree to which the horizontal semicircular canals are responsive, as well as how symmetric the responses are between the patient left and right ear [38, 39].

Nijmegen questionnaire for hyperventilation complaints. Is a screening tool to detect patients with hyperventilation complaints that could benefit from breathing regulation through capon graphic feedback[40].

### EEG data collection

EEG data was obtained as a standard procedure. For the patients with balance complaints, the EEG was recorded in a symptom free interval between episodes. Recordings were obtained in a fully lighted room with each participant sitting upright on a small but comfortable chair. The actual recording lasted approximately five minutes. The EEG was sampled using Mitsar-201 amplifiers (NovaTech http://www.novatecheeg.com/) with 19 electrodes placed according to the standard 10–20 International placement (Fp1, Fp2, F7, F3, Fz, F4, F8, T7, C3, Cz, C4, T8, P7, P3, Pz, P4, P8, O1, O2), analogous to what was done in the normative group. Impedances were checked to remain below 5 kΩ. Data was collected with eyes closed (sampling rate = 500 Hz, band passed 0.15-200Hz). Off-line data was resampled to 128 Hz, band-pass filtered in the range 2–44 Hz and subsequently transposed into Eureka! Software [41]. The data was then plotted and carefully inspected for manual artifact-rejection. All episodic artifacts including eye blinks, eye movements, teeth clenching, body movement, or ECG artifact were removed from the stream of the EEG. Average Fourier cross-spectral matrices were computed for frequency bands delta (2–3.5 Hz), theta (4–7.5 Hz), alpha1 (8–10 Hz), alpha2 (10-12Hz), beta1 (13–18 Hz), beta2 (18.5–21 Hz), beta3 (21.5–30 Hz), and gamma (30.5–44 Hz). These frequency bands are based on previous research in tinnitus [42–45].

### Source localization

Standardized low-resolution brain electromagnetic tomography [46] was used to estimate the intracerebral electrical sources that generated the seven group blind source separation components. As standard procedure, a common average reference transformation [46] is performed before applying the sLORETA algorithm. sLORETA computes electric neuronal activity as current density (A/m^2^) without assuming a predefined number of active sources. The solution space used in this study and associated lead field matrix are those implemented in the LORETA-Key software (freely available at http://www.uzh.ch/keyinst/loreta.htm). This software implements revisited realistic electrode coordinates [47] and the lead field produced by Fuchs et al. applying the boundary element method on the MNI-152 (Montreal neurological institute, Canada) [48]. The sLORETA-key anatomical template divides and labels the neocortical (including hippocampus and anterior cingulated cortex) MNI-152 volume in 6,239 voxels of dimension 5 mm^3^, based on probabilities returned by the Demon Atlas [49]. The co-registration makes use of the correct translation from the MNI-152 space into the Talairach and Tournoux space.

### Lagged phase coherence

Coherence and phase synchronization between time series corresponding to different spatial locations are usually interpreted as indicators of “connectivity”; however, any measure of dependence is highly contaminated with an instantaneous, non-physiological contribution due to volume conduction [50]. Pascual-Marqui, introduced new measures of coherence and phase synchronization taking into account only non-instantaneous (lagged) connectivity, effectively removing the confounding factor of volume conduction [51]. As such, this measure of dependence can be applied to any number of brain areas jointly, i.e., distributed cortical networks, whose activity can be estimated with sLORETA. Measures of linear dependence (coherence) between the multivariate time series are defined. The measures are non-negative, and take the value zero only when there is independence and are defined in the frequency domain: delta (2–3.5 Hz), theta (4–7.5 Hz), alpha (8-12Hz), low beta (13–21 Hz), high beta (21.5–30 Hz), and gamma (30.5–44 Hz). Based on this principle lagged linear connectivity was calculated. Time-series of current density were extracted for different regions of interest using sLORETA. Power in all 6,239 voxels was normalized to a power of 1 and log transformed at each time point. Region of interest values thus reflect the log-transformed fraction of total power across all voxels and do so separately for specific frequencies. Regions of interest selected were BA43, BA7, BA8, BA40, BA11, and BA24, corresponding respectively to the left and right vestibular cortex, left and right precuneus, left and right frontal eye field, left and right intraparietal sulcus, left and right orbitofrontal cortex, and the dorsal anterior cingulate cortex. These ROIs were defined based on previous brain research on vestibular symptoms [22, 52, 53].

### Alpha-gamma nesting

Alpha-gamma nesting is an effective way of communicating between cortically distant areas [54]. To examine if this alpha-gamma nesting is present in patients with chronic symptoms of vertigo, the alpha-gamma nesting is calculated in the left and right vestibular cortex and left and right frontal eye fields by alpha lagged phase synchronization. Alpha-gamma nesting was computed as follows: first, the time series for the *x*, *y*, and *z* components of the sLORETA current for the left and right vestibular cortex, as well as, the left and right frontal eye fields were obtained. Next, it was filtered in the alpha (8–12 Hz) and gamma (30–44 Hz) frequency band-pass regions. Those are the time series of the current in the three orthogonal directions in space. In each frequency band and for each ROI, a principle component analysis was computed, and the first component was retained for alpha and gamma. The Hilbert transform was then computed on the gamma component and the signal envelope retained. Finally, a Pearson correlation between the alpha component and the gamma envelope was computed.

### Statistical analysis

Descriptive statistics were calculated from 23 patients with balance complaints who completed two questionnaires. Overall participants’ demographics and scores were reported as mean (*M*) ± and standard deviation (*Sd*) as appropriate. Questionnaire scores were measured at baseline only, and served as a primary outcome parameter for patients with chronic symptoms of vertigo.

sLORETA was used to identify potential differences in the brain neural activity between patients with chronic symptoms of vertigo, and healthy controls. sLORETA performs voxel-by-voxel (comprising 6239 voxels) between groups’ comparison for the different frequency bands. The sLORETA images were created using nonparametric statistical analyses (SnPM) for each contrast, employing a t-statistic for unpaired groups with a correction for multiple comparisons (*p* < 0.05). Since this method is nonparametric in nature, it does not assume normal distribution and promptly accounts for the multiple comparison problem [55]. In addition, differences in the log-transformed current density were computed, using a MANOVA for all ROIs and all frequency bands. Using the correlation toolbox on sLORETA, a correlation analyses was computed to identify associations between the connectivity correlation and the VAS measures of intensity and discomfort as well as the DHI. Finally, correlations between alpha-gamma nesting and the VAS measures of intensity for the left and right frontal eye field, as well as the left and right vestibular cortex were computed.

## Results

### Behavioral measurements

Average scores of patients with chronic symptoms of vertigo were computed for their VAS measures of intensity (*N* = 23; *M* = 6.26; *Sd* = 2.30) and discomfort (*N* = 23; *M* = 6.89; *Sd* = 2.26), in addition to the DHI (*N* = 23; *M* = 50.52; *Sd* = 16.14). A Pearson correlation was computed and revealed a significant positive association between the VAS measures of intensity and discomfort (*N* = 23; *r* = .87, *p* < .001). A significant positive association was also identified for the VAS measure of intensity (*N* = 23; *r* = .71, *p* < .001), the VAS measure of discomfort (*N* = 23; *r* = .52, *p* = .02), and DHI. No significant association was identified between the duration of the chronic symptoms of vertigo attack, the VAS measure of intensity (*N* = 14; *r* = .19, *p* = .50), and the VAS measure of discomfort (*N* = 14; *r* = .10, *p* = .71). Similarly, no significant association was found for the VAS measure of intensity (*N* = 14; *r* = .13, *p =* .64) and the VAS measure of discomfort (*N* = 14; *r* = .05, *p =* .87) with the frequency of the symptoms. A whole brain group comparison between patients with chronic vestibular symptoms and healthy subjects

**Fig 1:**
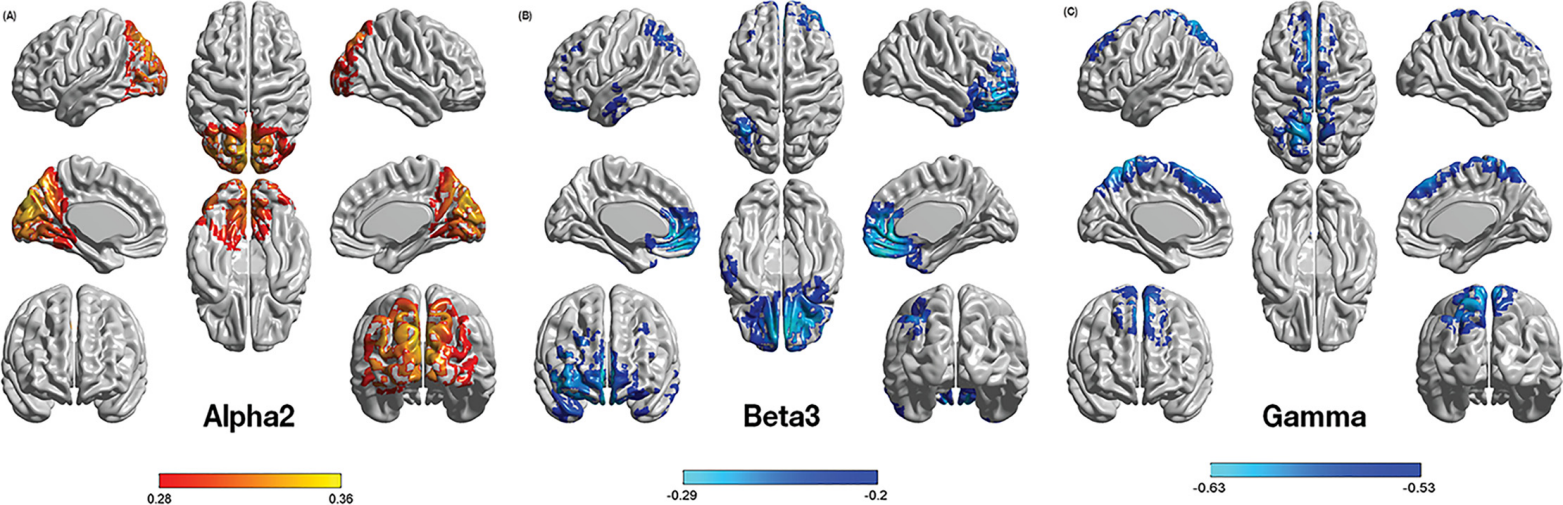
A whole brain group comparison between patients with chronic vestibular symptoms and healthy subjects. A comparison of source analyzed resting state brain activity between patients with chronic symptoms of vertigo and healthy subjects revealed: (A) a significant increase (in red) of alpha2 activity in the posterior cingulate cortex extending into the parahippocampal area, precuneus, and cuneus regions, (B) a decrease (in blue) for beta3, activity predominant in the ventral medial prefrontal cortex/pregenual anterior cingulate cortex, subgenual anterior cingulate cortex and the medial orbitofrontal cortex,as well as the left anterior midtemporal area and the dorsal attention network which incorporates regions of the superior parietal area and dorsolateral prefrontal cortex). As for gamma, figure (C) shows decreased (in blue) for gamma frequency band, mostly present in the pre-supplementary motor area extending into the frontal eye fields as well as the precuneus.

A comparison between patients with chronic symptom of vertigo and healthy subjects revealed a significant increase for the alpha2 frequency band (*F* = .28, *p* < .05) and reduced activity for the beta3 (*F* = -.20, *p* < .05) and gamma (*F* = -.55, *p* < .01) frequency bands. For alpha2, increased activity is found in the posterior cingulate cortex extending into the parahippocampal area, precuneus, and cuneus (Fig 1A). For beta3 decreased activity is localized predominantly in the ventral medial prefrontal cortex/pregenual anterior cingulate cortex, subgenual anterior cingulate cortex, and the medial orbitofrontal cortex, however, there is also decreased beta3 activity in the left anterior midtemporal area and the dorsal attention network (superior parietal area and dorsolateral prefrontal cortex) (Fig 1B). For the gamma frequency band, decreased activity is apparent in the pre-supplementary motor area extending into the frontal eye fields as well as the precuneus (Fig 1C). No significant results were obtained for the delta, theta, alpha1, beta1, and beta2 frequency bands.

### Whole brain correlation analysis with intensity

A correlation analysis between the VAS measure of intensity and brain activity revealed a significant correlation for delta (r = -.59, *p* < .001 & r = .69, *p* < .001), theta (r = -.49, *p* < .01 & r = .69, *p* < .001), alpha1 (r = .40, *p* < .05), alpha2 (r = .36, *p* < .05), beta1 (r = .45, *p* < .05), beta2 (r = .45, *p* < .05), beta3 (r = .45, *p* < .05) and gamma frequency bands (r = -.69, *p* < .001 & r = .60, *p* < .001).

For the delta frequency band, a negative correlation was identified with the frontal eye fields and a positive correlation with the subgenual anterior cingulate cortex, anterior medial temporal cortex, insula, and vestibular cortex.

For the theta frequency band, a negative correlation was identified with the precuneus/cuneus and a positive correlation with the subgenual anterior cingulate cortex, anterior medial temporal cortex, insula, and vestibular cortex.

**Fig 2:**
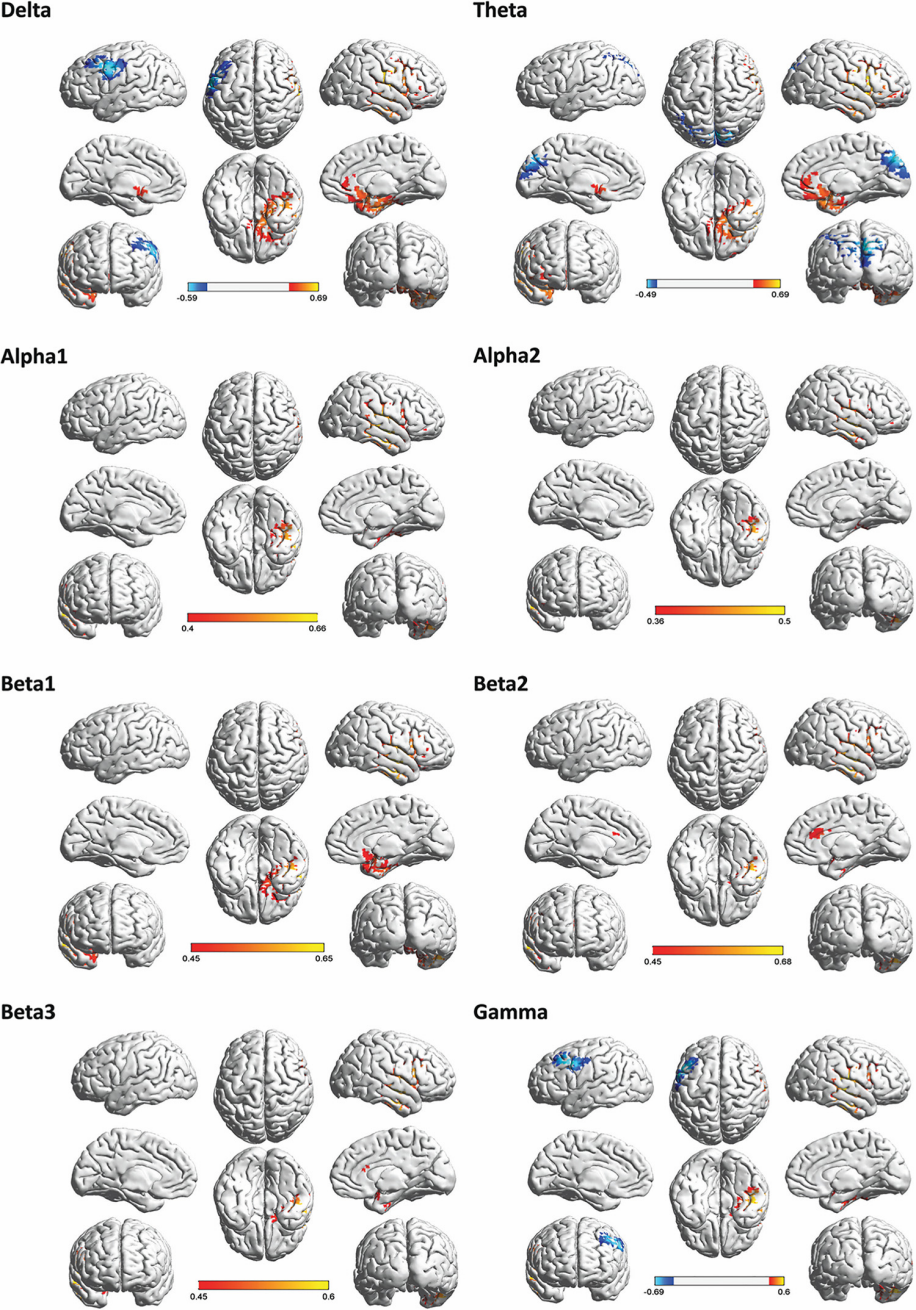
Correlation between brain frequency band activity and the VAS measure of intensity. There was a negative correlation between delta activity and the VAS measure of intensity (*r* = -.59) in the frontal eye fields, while delta activity correlated positively (*r* = .69) with the subgenual anterior cingulate cortex, anterior medial temporal cortex, insula, vestibular cortex, and the intensity of chronic symptoms of vertigo. As for theta activity a negative correlation (*r* = -.49) between the precuneus/cuneus, and a positive correlation (*r* = .69) with subgenual anterior cingulate cortex, anterior medial temporal cortex, insula, vestibular cortex, and the VAS measure of intensity was obtained. Alpha1 (*r* = .40), alpha2 (*r* = .36), beta1, beta2, and beta3 (*r* = .45) were positively correlated with the VAS measure of intensity and activity in the posterior insula, the subgenual anterior cingulate cortex, anterior medial temporal cortex, and the dorsal anterior cingulate cortex, respectively, As for gamma, a negative correlation (*r* = -.69) was identified for the frontal eye fields, and a positive correlation (*r* = .60) for the posterior insula extending into the vestibular cortex.

For the alpha1, alpha2, beta1, beta2, and beta3 frequency bands, the VAS measure of intensity is correlated positively with the posterior insula extending into the vestibular cortex. In addition, for the beta1 frequency a positive correlation was obtained with the subgenual anterior cingulate cortex and anterior medial temporal cortex, while for the beta2 and beta3 frequency bands, a positive correlation was found with the dorsal anterior cingulate cortex. For the gamma frequency band, a negative correlation was identified with the frontal eye fields and a positive correlation was revealed with the posterior insula extending into the vestibular cortex. Fig 2 shows a summary of the obtained results. A correlation analysis with DHI and VAS measure of discomfort obtained no significant results for the delta, theta, alpha1, alpha2 beta1, beta2, beta3, and gamma frequency bands.

### ROI analysis

**Fig 3:**
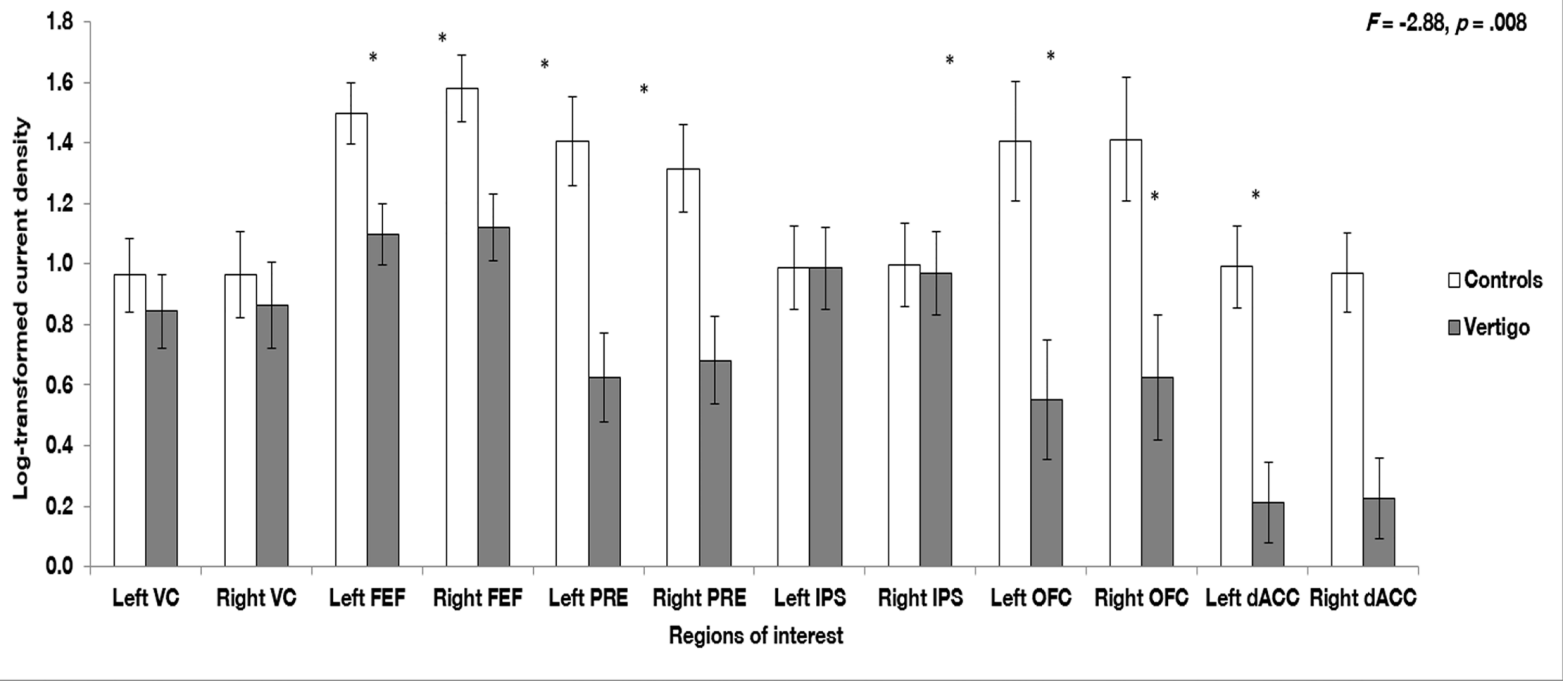
Region of interest (ROI) analysis. A one-way MANOVA analysis of variance showed a significant difference for the left and right frontal eye field (*p* = .007, *p* = .005), left and right precuneus (*p* = .00, *p* = .003), left and right orbitofrontal cortex (*p* = .004, *p* = .009), and the left and right dorsal anterior cingulate cortex (*p* < .001). The left and right vestibular cortex, as well as the left and right intra-parietal sulcus yielded no significant effects. Error bars designate standard errors, and a * indicate a significant difference.

A one-way MANOVA analysis of variance was conducted for each of the eight frequency bands separately. Results from MANOVA demonstrated a significant effect for the gamma frequency band and the left frontal eye field (*F* = 7.93, *p* = .007), right frontal eye field (*F* = 8.64, *p* = .005), left precuneus (*F* = 13.90, *p* = .001), right precuneus (*F* = 9.56, *p* = .003), left orbitofrontal cortex (*F* = 9.26, *p* = .004), right orbitofrontal cortex (*F* = 7.38, *p* = .009), left dorsal anterior cingulate cortex (*F* = 17.11, *p* < .001), and the right dorsal anterior cingulate cortex (*F* = 16.12, *p* < .001). No significant effect was obtained between gamma and the left vestibular cortex (*F* = .26, *p* = .61), right vestibular cortex (*F* = .47, *p* = .54), left intrapartiel sulcus (*F* = .00013, *p* = .97), or right intrapartiel sulcus (*F* = .01, *p* = .92). See Fig 3 of log-transformed current density for gamma frequency band activity with the different regions of interest. No significant differences were obtained for the delta, theta, alpha1, alpha2 beta1, beta2, and beta3 frequency bands.

In addition, correlation analyses were performed between the different ROIs and the VAS measure of intensity and discomfort, as well as the DHI. There was a significant negative association between the right precuneus and the DHI (*r* = -.42, *p* = .03). However, no significant associations were identified for any other ROIs with the VAS measure of intensity, the VAS measure of discomfort, or DHI.

### Functional connectivity analysis

**Fig 4:**
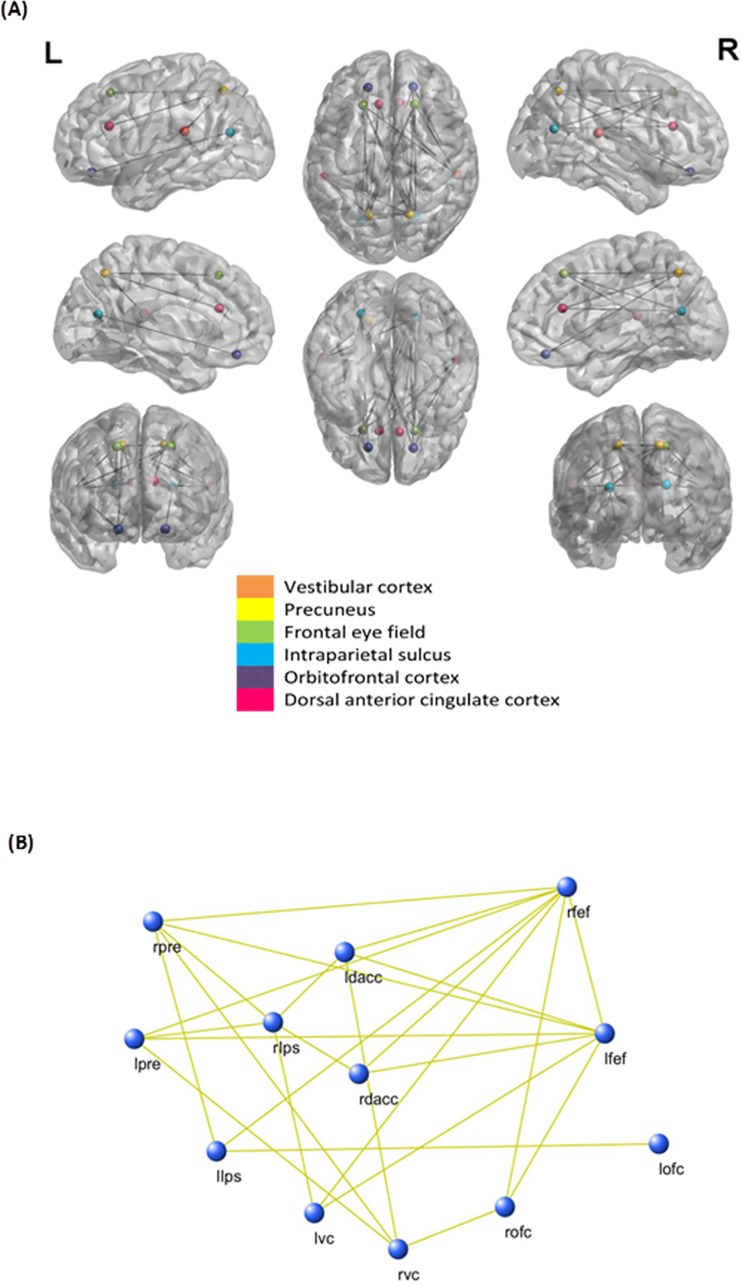
Functional connectivity analysis. Functional connectivity as measured by lagged phase synchronization for the gamma frequency band. (A) A decreased lagged phase synchronization is found for gamma between the vestibular cortex, precuneus, frontal eye field, intra-parietal sulcus, orbitofrontal cortex, and the dorsal anterior cingulate cortex. (B). Schematic representation of the interactions and connections between the different region of interests.

A comparison between patients with chronic symptoms of vertigo and healthy subjects for the functional connectivity analysis (lagged phase synchronization) yielded a significant difference (*p* < .05) for the gamma frequency band (Fig 4A & 4B). In general, reduced lagged phase coherence could be found for gamma band activity in the vestibular cortex, precuneus, frontal eye field, intra-parietal sulcus, orbitofrontal cortex, and the dorsal anterior cingulate cortex.

### Connectivity correlation analysis

A correlation analysis with brain functional connectivity and independently the VAS measure of intensity, the VAS measure of discomfort, and DHI obtained no significant results for the delta, theta, alpha1, alpha2 beta1, beta2, beta3, and gamma frequency bands.

### Alpha-gamma nesting and VAS measure of intensity

**Fig 5:**
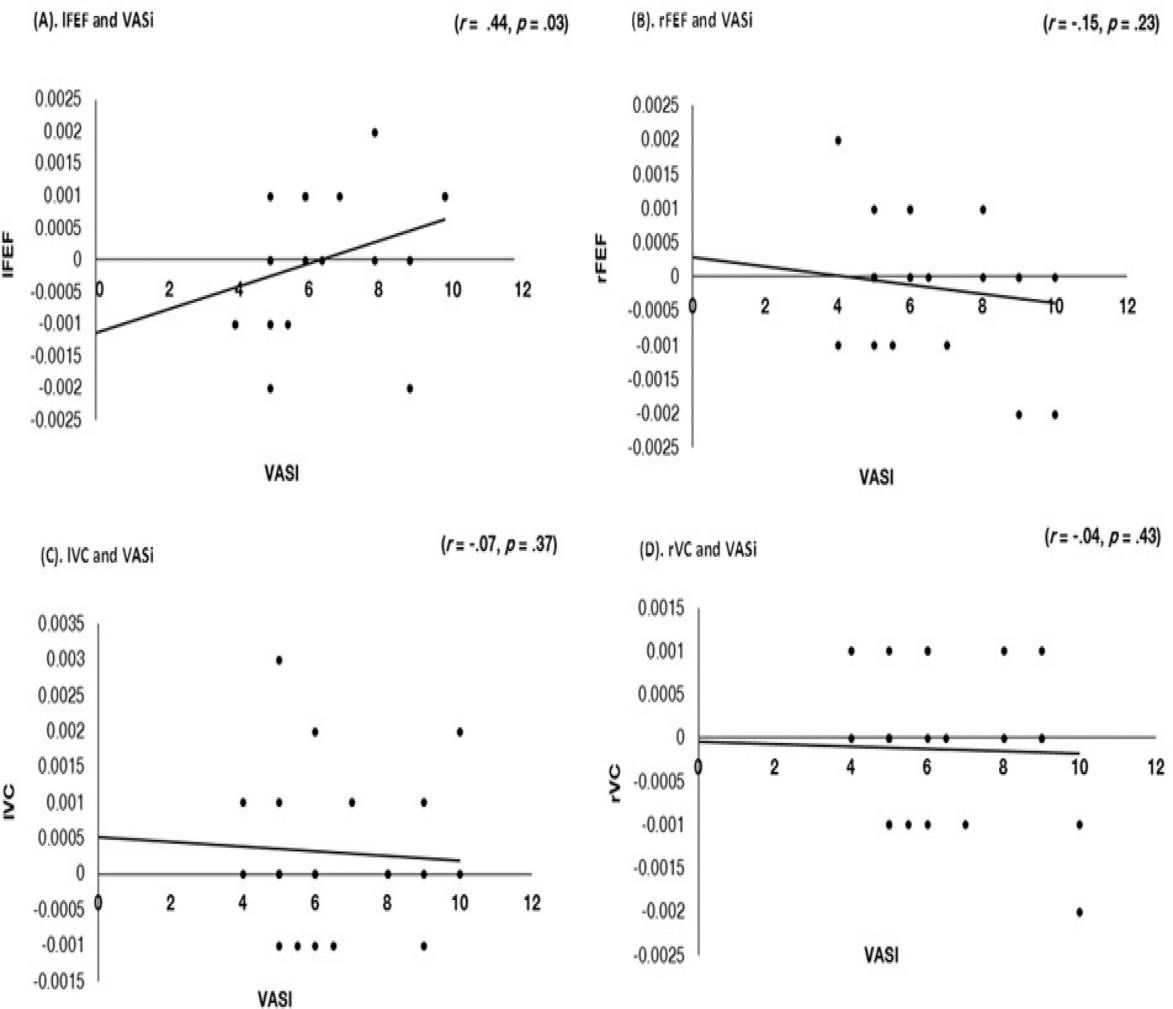
Correlation of alpha-gamma cross-frequency coupled oscillations (= nesting). A correlation of the frontal eye field (FEF), the vestibular cortex (VC) and the visual analogue scale (VAS) measure of intensity. (A) shows a positive correlation between left FEF (lFEF) and VAS measure of intensity (*r* = .44), suggesting as nesting of alpha-gamma in the left FEF increased, measures of VAS intensity increased. There was no association between nesting of alpha-gamma for (B) right FEF (rFEF), (C) left VC (lVC), and (D) right VC (rVC) and the VAS measure of intensity.

There was a significant positive correlation between alpha-gamma nesting for the left frontal eye field with the VAS measure of intensity (*r* = .44, *p* = .03). No association can be identified between alpha-gamma nesting and the right frontal eye field (*r* = -.15, *p* = .23), left vestibular cortex (*r* = -.07, *p* = .37), right vestibular cortex (*r* = -.04, *p* = .43) and the VAS measure of intensity (Fig 5).

## Discussion

The main objective of this study was to characterize the differences in resting brain activity and functional connectivity as measured by source analyzed EEG in patients with chronic symptoms of vertigo compared to healthy subjects, with data recorded in a symptom free interval, looking for a neural signature characteristic for proneness to develop balance complaints. Proneness to vestibular symptoms is related to increase alpha and reduced beta-gamma activity in multiple areas of the brain, which have been linked to vestibular function. In addition, the amount of balance symptoms (intensity) is correlated with the amount of electrical activity in some of these areas during a free symptoms resting state. This was not the case for the amount of discomfort perceived by the chronic symptoms of vertigo spells. The three main findings of this study are: 1) the localization of reduced delta and gamma band activity within the left frontal eye field, 2) hypoconnectivity between different vestibular brain regions, and 3) increased nesting of alpha-gamma oscillations within the left frontal eye field. Since our recordings were performed in the absence of a balance complaint episode, we assume that the findings demonstrate a neural signature for chronic balance symptom proneness, and support the potential of EEG-based measures of cortical activity and connectivity as biomarkers for chronic vestibular symptom proneness.

### Brain activity changes

#### Alpha activity

The comparison between resting state activity in patients with chronic symptoms of vertigo and healthy non-vertiginous controls revealed increased alpha2 frequency band activity in the posterior cingulate cortex extending into the precuneus/cuneus and parahippocampus. Recent studies have demonstrated the role of alpha activity in attentional cognitive processes [56–59], so the findings of this study—increased alpha activity within the cingulate cortex, the precuneus/cuneus and the parahippocampal area—are somehow to be anticipated since these regions are known to be anatomically connected and presumed to play a central role in supporting and regulating the focus of cognition, attention, consciousness, and the processing of basic visual information [60–64]. Furthermore, some evidence suggests that alpha oscillations inhibit task-irrelevant neural representations [57, 65]. Consequently, a key role for alpha appears to be as an attentional suppression mechanism when objects or features need to be specifically disregarded [66]. Giving the compelling accumulation of evidences pointing toward an active role of alpha in attention suppression and the processing of ongoing visual information [67, 68], the observed increase in alpha activity reported in our study, could also be a representation of an attentional disengagement action, which we hypothesize to manifest a similar internal inhibitory mechanism.

#### Beta activity

In addition, reduced beta3 activity was obtained that was mostly localized to the pregenual cingulate cortex, subgenual anterior cingulate cortex, and the medial orbitofrontal cortex in patients with chronic symptoms of vertigo. The pregenual anterior cingulate cortex might be a non-specific input-suppressing area. It is part of the pain inhibitory antinociceptive descending pathway [69, 70], which is deficient in general pain syndromes such as fibromyalgia [71]. Furthermore, the same pregenual area is involved in suppression of aggressive spells and is deficient in people exhibiting aggressive behavior [72]. The same area now seems to be deficient in patients with chronic symptoms of vertigo, which might be the vestibular analogue to other conditions such as tinnitus and pain, which share a common underlying pathophysiology [73].

#### Gamma activity

A reduced activity was also shown for the gamma frequency band in the pre-supplementary motor area extending to the frontal eye field in patients with chronic symptoms of vertigo. Gamma activity seems to reflect prediction error processing [74]. This could be hypothesized to reflect changes in vestibular input that are less detected and thus less processed by the frontal eye field. The frontal eye field and the orbitofrontal cortex are among the brain regions involved in self-awareness, attention, and perception for all senses [75–81].

The results of the current study also show that electrophysiological changes in the posterior insula correlate with the perceived intensity of the balance complain for all frequency bands (i.e., delta, theta, alpha1, alpha2, beta1, beta2, beta3, and gamma). Research already suggested that the posterior insula contains the primary vestibular cortex, as vestibular stimuli seem to activate that region, mostly ipsilaterally [15, 82]. Accordingly, the posterior insula was considered a critical component to the experience of chronic symptoms of vertigo [83]. Our findings are also in line with previous functional imagining studies that support parts of the insula, clusters in the temporo-parietal cortex, lateral and medial premotor cortex, orbitofrontal cortex, and the anterior cingulate were involved in a network that was asymmetrically activated in patients with vestibular dysfunctions [15, 84–86]. Moreover, abnormal activity in the insula and cingulate cortex was linked to emotional reaction to distress in other pathologies [87–89]. This implies that these brain regions are parts of a non-specific distress network [73, 90]. Similarly, the current study demonstrates a link between chronic vestibular symptoms of vertigo intensity and increased electrophysiological (i.e., delta, theta, alpha1, alpha2, beta1, beta2, beta3, and gamma) activity in the insula, subgenual, and dorsal anterior cingulate cortex, which might be suggestive of a dynamically active network, similar to that proposed in tinnitus research [73, 90], and might correlate with different aspects of the chronic symptoms of vertigo experience.

No correlation was found between the behavioral measures of discomfort, dizziness handicap, and electrophysiological changes, with the exception of a negative correlation between the DHI and right precuneus activity. The lack of consistency between outcomes of physiological and behavioral measures of chronic symptoms of vertigo is somehow predictable. That is, generally poor agreement has been reported between modality-specific subjective-report of the magnitude of the chronic symptoms of vertigo and the actual responses of objective measures [91, 92], suggesting their fundamental differences are analogous to what has been described in the auditory system [93].

#### Relationship between alpha-gamma nesting and VAS measure of intensity

There is evidence pointing toward the key role the posterior insula plays in the processing of sensorimotor information, as it assumed to represent a main region of the human vestibular cortical network [15, 94]. This was supported by the findings of this study as changes in the posterior insula correlated with measures of intensity for all frequency bands.

As in otherwise, no systematic empirical research exists addressing the question of alpha-gamma cross frequency coupling in relation to measure of intensity in patients with balance complains. Furthermore, to the best of our knowledge, this study is the first to address such relationship. Accordingly, our finding of no association between nesting of alpha-gamma and measure of intensity for the right frontal eye field, and the left and right vestibular cortex, although substantial, it requires further specific examination.We can only hypothesize based on findings of recent research [95–97] that pointed toward a disassociation between vestibular information and information provided through different sensory modalities in conditions where the vestibular system is affected as a possible compensatory mechanism, that the observed disconnection between the nesting of alpha-gamma in the vestibular cortex and the VAS measure of intensity in this study, could manifest a similar mechanism.

#### Brain connectivity changes

The current study has found reduced functional connectivity for gamma between the vestibular cortex, precuneus, frontal eye field, intra-parietal sulcus, orbitofrontal cortex, and the dorsal anterior cingulate cortex. Gamma activity is known to modulate cognitive mechanisms and has been hypothesized to support communication between different anatomically distributed cortical areas. [98–100]. Gamma activity is usually focally restricted [101] and waxes and wanes [99]. It has been shown that gamma is nested by means of cross-frequency coupling with theta [54] and alpha [99, 102], both of which function as carrier waves, binding segregated focal gamma activity into one unified percept. To examine this, we looked at the nesting of alpha-gamma oscillations within the left and right frontal eye field. Studies indicated that the frontal eye field is involved in visual attentional control via modulation of alpha activity [59, 103–107]. This is a mechanism believed to be under a top-down attentional control [108] which permits modular processes to be temporarily available to consciousness [99]. A recent investigation demonstrated that both the left and right frontal eye fields are causally involved in the attentional top-down control of anticipatory alpha in the contralateral visual system [107], although an earlier study showed that the left but not the right frontal eye field is selectively and significantly involved in short-term storage of location [109]. In the light of these results, the left frontal eye field is implied to play a key role in short-term memory storage of spatial position, which is not always related to eye movement so can be considered functionally as a visual system sensory area [58, 107, 109]. The findings of the current study demonstrate increased alpha-gamma oscillations within the left frontal eye field, which is supported by findings of previous investigations showing that alpha activity provides a clocking mechanism which controls neural processing reflected by activity in the gamma band, creating a sequence of perceptual cycles [58, 62, 110], as perception is linked to various visual illusions created by rhythmic oscillatory modulation of alpha activity [111–113]. Moreover, it was suggested that the visual system is inhibited during most of the alpha cycle, whereas a burst of gamma activity at a specific alpha phase reflects a window of excitability [59]. To that extent, alpha oscillations have been proposed to represent functional inhibition of the human visual system [110], providing a mechanism for prioritizing and ordering unattended visual input based on their relevance, whereas gamma oscillations keep opposing unattended representations apart in time [58]. This type of increased alpha-gamma oscillation will be part of a compensating mechanism of the brain-reduced aptitude to localize the self in space and context. As a result, the system heightens visual attention to establish a better percept of the individual contextual localization of his/her surroundings.

## Conclusion

The finding of this study hypothesize that chronic symptoms of vertigo might be related to altered brain activity in widespread vestibular areas associated with hypoconnectivity and is also possibly linked to deficient vestibular suppression. Furthermore, increased alpha-gamma nesting is likely part of a compensatory mechanism as the brain attempts to form a better contextual localization of the individual surrounding when perception to context and space seem to decline.
